# Fluorescence lifetime imaging and electron microscopy: a correlative approach

**DOI:** 10.1007/s00418-022-02094-0

**Published:** 2022-03-10

**Authors:** Johannes G. Wieland, Nilanjon Naskar, Angelika Rück, Paul Walther

**Affiliations:** 1 Central Facility for Electron Microscopy, Ulm University, 89081 Ulm, Germany; 2 Core Facility Confocal and Multiphoton Microscopy, Ulm University, 89081 Ulm, Germany

**Keywords:** Fluorescence lifetime imaging microscopy (FLIM), Fluorescence lifetime imaging and electron microscopy (FLEM), Correlative light- and electron microscopy (CLEM), STEM tomography, NADH, Cell metabolism

## Abstract

Fluorescence lifetime imaging microscopy (FLIM) allows the characterization of cellular metabolism by quantifying the rate of free and unbound nicotinamide adenine dinucleotide hydrogen (NADH). This study delineates the correlative imaging of cells with FLIM and electron microscopy (EM). Human fibroblasts were cultivated in a microscopy slide bearing a coordinate system and FLIM measurement was conducted. Following chemical fixation, embedding in Epon and cutting with an ultramicrotome, tomograms of selected cells were acquired with a scanning transmission electron microscope (STEM). Correlative imaging of antimycin A-treated fibroblasts shows a decrease in fluorescence lifetime as well as swollen mitochondria with large cavities in STEM tomography. To our knowledge, this is the first correlative FLIM and EM workflow. Combining the high sensitivity of FLIM with the high spatial resolution of EM could boost the research of pathophysiological processes involving cell metabolism, such as cancer, neurodegenerative disorders, and viral infection.

## Introduction

Fluorescence lifetime imaging microscopy (FLIM) of nicotinamide adenine dinucleotide hydrogen (NADH) has proven to be a valuable tool for the characterization of cell metabolism and mitochondrial function with a broad range of bioenergetics applications (Schaefer et al. 2019). However, being limited to the spatial resolution of light microscopes, FLIM can not be used to investigate the cellular ultrastructure. Therefore, this study aims to combine FLIM with electron microscopy. The underlying assumption was that FLIM and EM would suit each other well, since FLIM measurement is carried out in unstained living cells in the native state. Therefore, in contrast to fluorescence microscopy of stained cells, no ultrastructural changes are introduced that could hamper EM imaging. In this study, a correlative FLIM and EM (FLEM) protocol was established and evaluated using imaging of antimycin A-treated fibroblasts. Such a protocol would help to improve the understanding of physiological processes in mitochondria as well as the mechanisms behind diseases involving cell metabolism. This is especially promising for diseases where FLIM is already established as a method to investigate pathomechanisms, like neurodegenerative diseases (Gomez et al. 2018), cancer (Kalinina et al. 2021), eye diseases, and cardiovascular disorders (Marcu 2012).

## Materials and methods

### FLIM

**Fig. 1:**
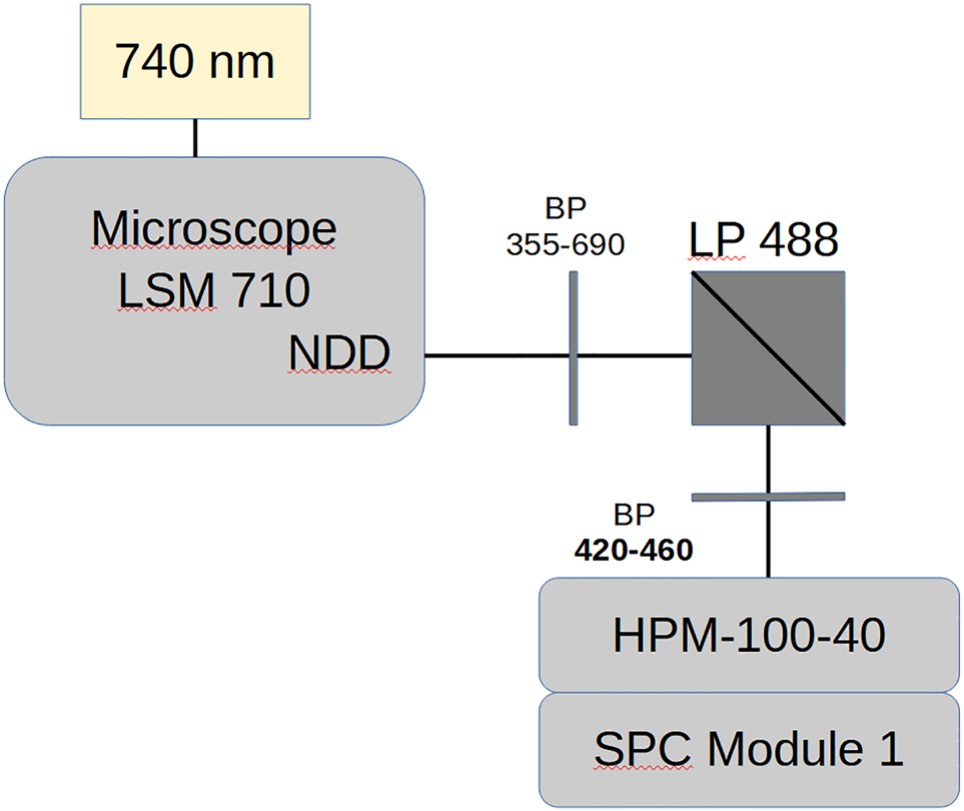
Setup of the FLIM system. Image was reproduced with courtesy to Dr. Sviatlana Kalinina, Core Facility Confocal and Multiphoton Microscopy, Ulm University

Human fibroblasts were cultivated on a µ-Slide 8 Well Grid-500 (ibidi) bearing a 10 × 10 coordinate system in each well. Cells were removed from the incubator and the medium was replaced with Tyrode’s buffer (135 mM NaCl, 5 mM KCl, 1 mM MgCl_2_, 1.8 mM CaCl_2_, 20 mM HEPES, 5 mM glucose, pH 7.4). Antimycin A-treated cells were incubated for 15 min before image acquisition and immediate fixation. FLIM measurement of NADH autofluorescence was conducted as described by Kalinina et al. (2021). In order to ensure that the region of interest imaged with FLIM could be located after preparation for electron microscopy, cells were selected at specific coordinates. Images were acquired using a Zeiss LSM 710 NLO laser scanning microscope equipped with a femtosecond pulsed Mai Tai AX HPDS titanium-sapphire laser (SpectraPhysics) with a repetition rate of 80 MHz and a tuning range of 690–1040 nm. The laser pulse for two-photon excitation was linked to a TCSPC system. Signal detection was accomplished through the non-descanned detection (NDD) port of the microscope with an HPM-100-40 hybrid detector system (Becker & Hickl GmbH). The full setup of the FLIM system is depicted in Fig. 1. FLIM images were acquired using the SPC64 software (Becker & Hickl GmbH) with a resolution of 512 × 512 pixels. Data analysis was performed using the SPCImage software version 8.1 (Becker & Hickl GmbH). The fluorescence decay curve of the NADH autofluorescence was fitted to an incomplete two-component exponential model:

1 $$I(t) = a_{1} e^{{{{ - t} \mathord{\left/ {\vphantom {{ - t} {\tau_{1} }}} \right. \kern-\nulldelimiterspace} {\tau_{1} }}}} + a_{2} e^{{{{ - t} \mathord{\left/ {\vphantom {{ - t} {\tau_{2} }}} \right. \kern-\nulldelimiterspace} {\tau_{2} }}}}$$

*I*(*t*) is the fluorescence intensity, *a*_1_ and *a*_2_ are the relative amplitudes, and τ_1_ and τ_2_ are the fluorescence lifetimes of the two components. Fixed lifetime values of τ_1_ = 400 ps and τ_2_ = 2500 ps were used to represent free and protein-bound NADH, respectively. Based on the results for each pixel with a binning factor of 1, a colormap was created where the fluorescence lifetime corresponds to a color legend.

### Chemical fixation

Following FLIM measurement, cells were chemically fixed (Bozzola 2014). Tyrode’s buffer was removed and replaced with 2.5% glutaraldehyde. Samples were then kept at 5 °C. For embedding, cells were washed three times with PBS (phosphate buffered saline) for 10 min each step; 2% osmium tetroxide in water was added for post-fixation. Samples were left for 2 h and then gradually dehydrated with 30%, 50%, 70%, and 90% propanol for 3 min each; 2% uranyl acetate dissolved in propanol was added and incubated at 37 °C for 30 min. Samples were washed with 100% propanol three times for 30 min each. Epoxy resin (Epon) in propanol was added at a ratio of 1:2 for 15 min, 1:1 for 30 min, and 2:1 for 1 h. Samples were then covered with 100% epoxy resin and left overnight, before being treated with fresh resin and left to polymerize at 60 °C for 48 h. The bottom of the ibidi µ-Slide was removed with a trim diamond (diatomeknives.com), leaving an imprint of the coordinate system on the Epon blocks. For TEM imaging, sections with a thickness of 70 nm were cut with a Leica Ultracut Microtome and mounted on copper slot grids. For STEM tomography, sections of 700 to 900 nm were cut with a 35° diamond knife (diatomeknives.com) and mounted on a glow discharged copper grid with parallel bars. A droplet of 10% (w/v) poly-l-lysine (Sigma Aldrich) in water was added. Sections were dried for 5 min at 37 °C. Then 15 μl of a solution containing 15 nm gold particles (Aurion.com) diluted 1:2 in water was applied to both sides of the sections to serve as fiducial markers for tomogram reconstruction. Finally, a 5 nm carbon coating was added on both sides with a Balzers BAF 300 electron beam evaporation device (opticsbalzers.com).

### TEM and STEM tomography

Samples were examined with a Jeol JEM 1400 transmission electron microscope to evaluate whether the cells imaged in FLIM could be located. Cells were then imaged with a Jeol JEM-2100F scanning transmission electron microscope at 200 kV acceleration voltage using the EM-Tools software (TVIPS, Tietz). A series of 97 bright-field images with ×200,000 magnification was acquired at tilt angles from − 72° to + 72° with 1.5° increment. Illumination time per image was 22 s. Each image had 1024 × 1024 pixels, with a pixel size of 2.74 nm. Tomogram reconstruction and segmentation was done with the IMOD package (Kremer et al. 1996), version 4.9.12.

## Results and discussion

**Fig. 2:**
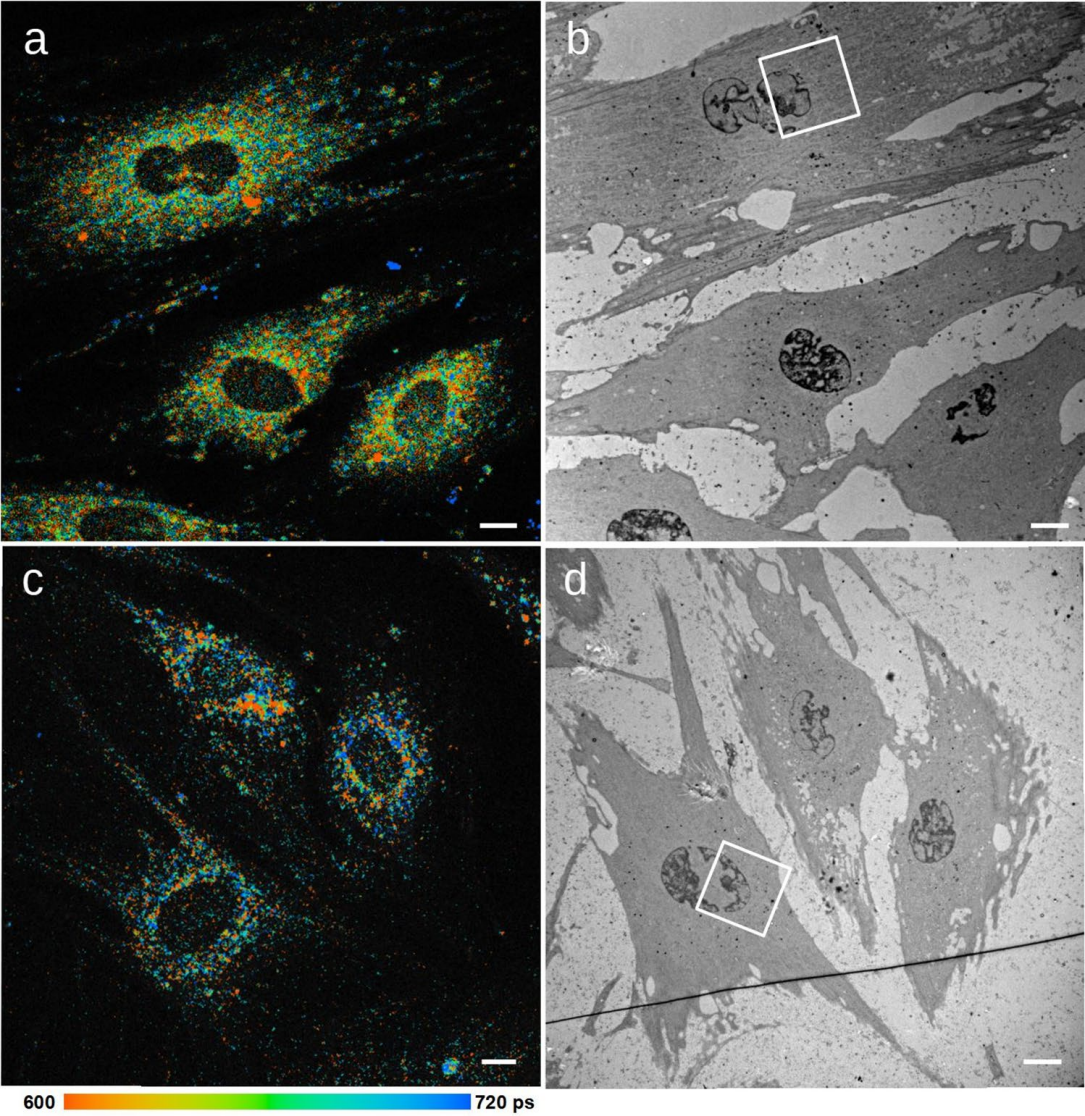
**a** Fluorescence lifetime image of human fibroblasts treated with antimycin A (false color). Compared to** c**, colors are shifted from blue to green and red. This indicates a shorter fluorescence lifetime τ_mean_ of NADH. **b** TEM image of the cells shown in a. **c** Fluorescence lifetime image of untreated human fibroblasts in false colors. Blue represents areas were a longer decay time τ_mean_ was detected. This corresponds to protein-bound NADH as found in mitochondria. **d** TEM image of the same cells after chemical fixation and embedding. The scalebar represents 10 μm in all four images, the color coding indicates τ_mean_

A number of advanced correlative light and electron microscopy approaches have been published. For an overview, see the book* Correlative Light and Electron Microscopy IV*, edited by Thomas Müller-Reichert and Paul Verkade (2021). However, as compared to other fluorescence microscopic approaches, FLIM of NADH stands out because it makes no use of fluorescent dyes but rather uses the intrinsic fluorescence of NADH in living cells of a defined physiological state. FLIM is extremely sensitive to slight adaptations that are convenient for subsequent EM preparation. The main difficulty in establishing a protocol for correlative FLIM and EM was to find a carrier system that would allow us to find the cells imaged in FLIM after sample preparation for EM. While this is a general problem in all CLEM applications, the high sensitivity of the FLIM system posed additional challenges. Chemical fixation proved successful because it can be done in a culture dish suitable for FLIM. The cells imaged in FLIM could be identified on the basis of their appearance on ultrathin sections in TEM (Fig. 2b, d) and their position relative to the coordinate system of the ibidi µ-Slide, which was visible as an imprint on the Epon block and the sections. On the basis of this information, the same cells could be located on tomography sections in STEM.

Correlative imaging of antimycin A-treated cells was chosen to evaluate the possibilities of FLEM. Antimycin A inhibits complex III of the respiratory chain and thereby leads to a lower complex V activity and an impaired mitochondrial energy metabolism. It shifts the ratio between intracellular protein-bound and free NADH and increases the contribution of free NADH. This is known to result in shorter decay times for NADH (Schaefer et al. 2019) in FLIM. As expected, the cells treated with antimycin A showed a shorter mean NADH lifetime (mainly green and red in Fig. 2a) than untreated cells (mainly blue in Fig. 2c).

**Fig. 3:**
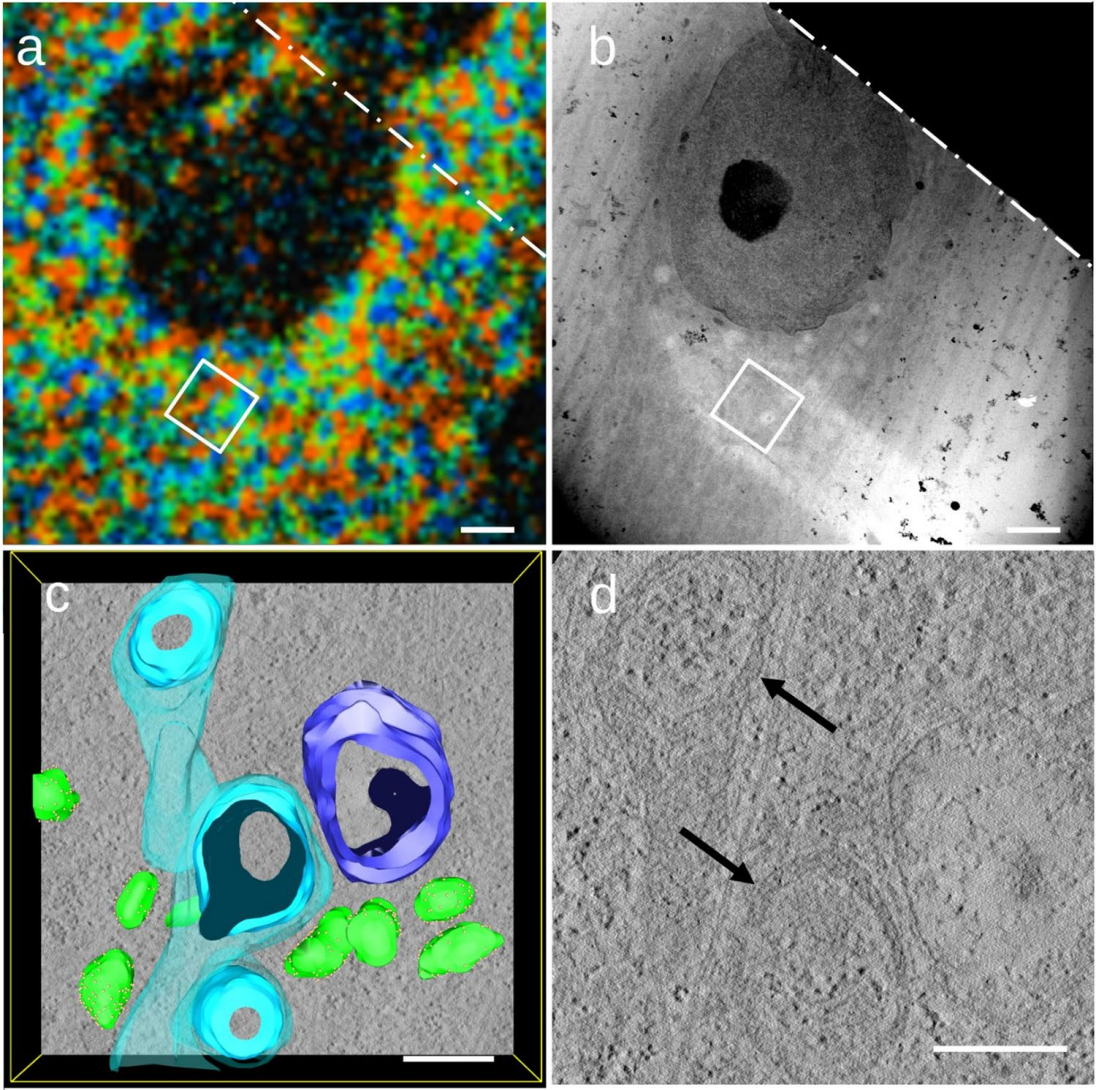
**a** FLIM image from the upper cell in Fig. 2a, b. The rectangle marks the rough position of the tomogram shown in **c**. **b** STEM overview of the same area. The white rectangle below the nucleus indicates where the tomogram from Fig. 3c was recorded. **c** is rotated 30° counterclockwise in comparison to the rectangle in Fig. 2a, b. **c** Segmentation of a tomogram of the upper cell from Fig. 2a, b. Rough ER (green) with ribosomes (yellow), a large vesicle (dark blue), and mitochondria (light blue) with a single cavity and no visible cristae. **d** Close-up of structurally altered mitochondria (black arrows) from Fig. 3c with large cavities. The scalebar represents 2 μm in** a** and** b** and 500 nm in **c** and **d**

**Fig. 4:**
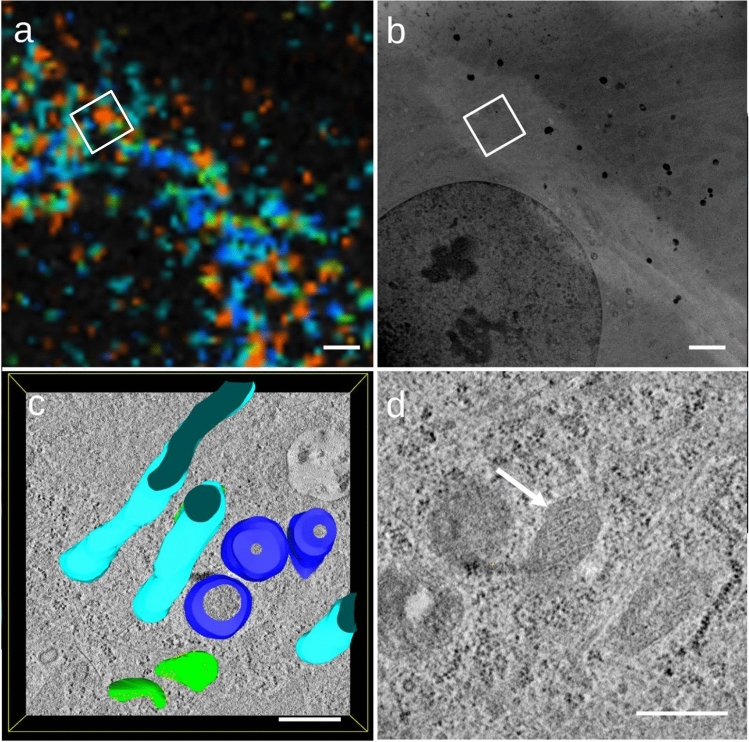
**a** FLIM image from the left cell in Fig. 2c, d. **b** STEM overview of the same area. The white rectangle above the nucleus indicates where the tomogram from Fig. 4c was recorded. **c** is rotated 120° clockwise in comparison to the rectangle in Fig. 4a, b. **c** Tomogram from the left cell from Fig. 2c, d showing unaltered mitochondria in untreated cells, as well as vesicles (dark blue) and rough ER (green). **d** Close-up of a mitochondrion (white arrow) with well-defined cristae from the untreated cell shown in **c**. The scalebar represents 2 μm in** a** and** b** and 500 nm in **c** and **d**

Correlation of FLIM and STEM tomography could be achieved by comparison of a close-up from FLIM and a STEM overview (Figs. 3a, b; 4a, b). One STEM tomogram of cells treated with antimycin A (Fig. 3c, d) shows a major change in the ultrastructure compared to untreated cells (Fig. 4c, d). Mitochondria (light blue in Figs. 3c, 4c) change their appearance from the tubular shape seen in untreated cells to a more compact form. They have no discernible cristae, but instead show large cavities that appear to be filled with cytoplasm (Fig. 3d). To our knowledge, the effects of antimycin A have not yet been studied by three-dimensional electron microscopic approaches in eucaryotic cells. Braet et al. (2003) used SEM imaging on antimycin A-treated rat hepatic sinusoidal endothelial cells, but not on a subcellular level. TEM imaging has been utilized in the context of autophagy (Elswood et al. 2021; Zhen et al. 2020), however with the main focus on autophagic membranes rather than mitochondrial ultrastructure. Elson et al. (1970) reported no ultrastructural changes caused by antimycin A in TEM imaging of protozoa. Tzung et al. (2001) and Hytti et al. (2019) observed swollen mitochondria in TEM images of antimycin A-treated cells which also showed evidence for a switch to glycolysis. Unfortunately, neither of those studies provides high magnification images of mitochondria, so a comparison with the results presented here is hard to accomplish. In this study, a possible correlation between shorter NADH decay time and a change in three-dimensional mitochondrial ultrastructure was observed. The STEM tomogram depicted in Fig. 3c shows three mitochondria with large cavities. The shorter fluorescence lifetime observed in FLIM correlates with free NADH which might accumulate in the cavities observed in the mitochondria. The lumen of the cavities shows a texturing similar to cytoplasm; however, no connection to the cytoplasm could be demonstrated in our data. Interestingly, a mitochondrion with large cavities has been reported by Marjan Huizing in a cell of a patient with a deficiency in adenine nucleotide transporter (published in Frey and Manella 2000, Fig. 6). Additionally, Huizing’s data shows numerous smaller vesicles inside the mitochondrion, a feature missing from antimycin A-treated fibroblasts presented here. The results presented here are based on single observation and further research is needed to confirm the effects of antimycin A on three-dimensional mitochondrial ultrastructure. Also, correlation of FLIM and EM images currently does not extend to the level of single mitochondria. Despite these limitations, our results might contribute to a refined model for the structural effects induced by antimycin A.

### Outlook: FLIM and EM imaging of high pressure frozen cells

Chemical fixation does not deliver optimal results in terms of sample preparation. Our current protocol suffers from poor visibility of lipid membranes (Figs. 3d, 4d). Sample preparation by high pressure freezing, however, is considered the gold standard for STEM tomography of thick specimens (Mielanczyk et al. 2014). Höhn et al. (2011) showed that it is particularly useful for the three-dimensional imaging of mitochondria, which makes it favorable for FLEM. We tested an approach for FLEM of high pressure frozen, freeze substituted cells. High pressure freezing, freeze substitution, and thin sectioning for TEM were carried out according to the protocol of Villinger et al. (2014) with cells cultivated on carbon-coated sapphire disks. However, the carbon layer caused strong reflections in FLIM and no images could be acquired. Cells grown on sapphire disks without carbon coating could not be embedded properly, because they would stick to the surface of the disks. Therefore, we decided to use focused ion beam scanning electron microscopy (FIB SEM), because, unlike STEM tomography, it does not rely on embedding and sectioning. Samples were prepared for FIB SEM by thin-layer plastification (Kizilyaprak et al. 2014) and imaged with an FEI Helios Nanolab 600 (Thermo Fisher). EM images could be acquired, but ultimately we could not achieve correlation. Characteristic landmarks such as the shape and position of nuclei were not visible and thus the EM images could not be correlated with FLIM data. This is likely an issue of missing experience rather than the method itself, since Kizilyaprak et al. (2014) demonstrated the visibility of these structures after thin-layer plastification. A starting point for further refinement of both protocols would be to improve the accuracy of correlation of FLIM and EM images. One well-established method in CLEM would be the use of markers consisting of fluorophores and heavy metals (Brown & Verkade 2010). Alternatively, software like CLEM*Site* (Serra Lleti et al. 2021) could be used to transfer stage positions of cells from the light microscope to a FIB SEM with high accuracy.

## Data Availability

All data will be made available upon reasonable request.
